# DNA barcode library of megadiverse Austrian Noctuoidea (Lepidoptera) – a nearly perfect match of Linnean taxonomy

**DOI:** 10.3897/BDJ.7.e37734

**Published:** 2019-08-07

**Authors:** Peter Huemer, Christian Wieser, Wolfgang Stark, Paul D.N. Hebert, Benjamin Wiesmair

**Affiliations:** 1 Tiroler Landesmuseen Betriebsges.m.b.H., Innsbruck, Austria Tiroler Landesmuseen Betriebsges.m.b.H. Innsbruck Austria; 2 Landesmuseum Kärnten, Klagenfurt am Wörthersee, Austria Landesmuseum Kärnten Klagenfurt am Wörthersee Austria; 3 , Trübensee, Austria Trübensee Austria; 4 Biodiversity Institute of Ontario, Guelph, Canada Biodiversity Institute of Ontario Guelph Canada

**Keywords:** Noctuoidea, Austria, COI gene, DNA barcoding, species identification, Lepidoptera

## Abstract

The aim of the study was to establish a nationwide barcode library for the most diverse group of Austrian Lepidoptera, the Noctuoidea, with 5 families (Erebidae, Euteliidae, Noctuidae, Nolidae, Notodontidae) and around 690 species. Altogether, 3431 DNA barcode sequences from COI gene (cytochrome c oxidase 1) belonging to 671 species were gathered, with 3223 sequences >500 bp. The intraspecific divergence with a mean of only 0.17% is low in most species whereas interspecific distances to the Nearest Neighbour are significantly higher with an average of 4.95%. Diagnostic DNA barcodes were obtained for 658 species. Only 13 species (1.9% of the Austrian Noctuoidea) cannot be reliably identified from their DNA barcode (*Setina aurita*/*Setina irrorella*, *Conisania leineri*/*Conisania poelli*, *Photedes captiuncula*/*Photedes minima*, *Euxoa obelisca*/*Euxoa vitta*/*Euxoa tritici*, *Mesapamaea secalella*/*Mesapamea secalis*, *Amphipoea fucosa*/*Amphipoea lucens*). A similarly high identification performance was achieved by the Barcode Index (BIN) system. 671 species of Austrian Noctuoidea, representing 3202 records with BINs, are assigned to a total of 678 BINs. The vast majority of 649 species is placed into a single BIN, with only 13 species recognised as BIN-sharing (including the barcode sharing species above). Twenty-one species were assigned to more than one BIN and have to be checked for cryptic diversity in the future.

## Introduction

With about 4070 species, Austria is one of the most diverse countries for Lepidoptera in Europe (Huemer 2013), only exceeded by few and usually much larger, mainly Mediterranean countries, particularly Spain, France, Italy, and Greece. Early studies of the Austrian fauna of Lepidoptera already date back to the Linnean period and include the famous „Systematisches Verzeichnis der Schmetterlinge der Wienergegend“ by Denis and Schiffermüller (1775), and continued for the last 250 years. The long lasting tradition of species delimitation in Lepidoptera (and other insects) was traditionally based on morphological features. This approach has recently dramatically changed with molecular methods, particularly DNA barcoding (Hebert et al. 2003b). This method of species identification by means of DNA barcode sequences is essentially based on the assumption of constant inter-specific divergences between sister species, even when considering intraspecific variation. This means that a single specimen of one species is grouped closer to the next specimen of the same species than to the next species, and there is no genetic overlap between the two species.

Within the last 10 years, more than 3000 species of Lepidoptera from the Austrian territory have been barcoded and the results of extensive inventories were published in parts (Huemer and Hebert 2015, Huemer and Hebert 2016). However, a complete nationwide analysis of species-rich groups at the superfamily or family level has so far been carried out only for the butterflies (Huemer and Wiesmair 2017). We now test barcode performance of a further large and representative superfamily of Austrian Lepidoptera, the Noctuoidea, with altogether five families, viz. Euteliidae, Erebidae, Noctuidae, Nolidae and Notodontidae, that cover ca. 690 species (Huemer 2013).

## Material and methods

### Sampling strategy

Voucher material was principally restricted to Austrian samples, with few exceptions of supplementing species known from the country but for which we failed to get sequence data. In order to study geographic variation we divided Austria in three major areas, which are concordant with political provinces and partially reflect biogeographic areas: a) North-Eastern Austria (Burgenland, Vienna, Lower Austria, Upper Austria), b) Southern Austria (Styria, Carinthia, East Tyrol), and c) Western Austria (Salzburg, North Tyrol, Vorarlberg). We then tried to obtain 4 specimens per species, with a minimum of one sample from each of the three areas. Similarly, the sought number of samples for regionally restricted species only present in one or two core areas was 4 specimens. Altogether, we selected 3431 specimens of Austrian Noctuoidea, including 63 specimens from 9 additional countries: Erebidae (647), Euteliidae (1), Noctuidae (2470), Nolidae (90) and Notodontidae (224). Taxonomy follows Huemer (2013), Witt and Ronkay (2011).

Recently collected samples were taken from various museum collections, particularly from Tiroler Landesmuseum Ferdinandeum (Innsbruck) (2000), Landesmuseum Kärnten (Klagenfurt) (523) and Niederösterreichisches Landesmuseum (St. Pölten, coll. Stark) (561), and from 23 additional institutional or private collections (348). Unfortunately, material of few species was not available in the necessary quality standards for DNA barcoding, viz. too old and likely degraded. Only in exceptional cases of very rare species we used such vouchers and tried to recover sequences with NGS protocols.

### DNA barcoding

DNA barcode sequences of the mitochondrial COI gene (cytochrome c oxidase 1) were obtained from 3431 specimens. DNA samples from dried legs were prepared according to prescribed standards using the high-throughput protocol of deWaard et al. (2008). Samples were processed at the Canadian Centre for DNA Barcoding (CCDB, Biodiversity Institute of Ontario, University of Guelph) to obtain DNA barcodes. DNA sequencing resulted in a barcode fragment of >500 bp for 3223 specimens belonging to 671 species, with 3160 sequences corresponding to the criteria of barcode compliance. 2962 sequences cover the full 658 bp, exceptionally with a high amount of ambiguous bases, particularly for the few sequences recovered with NGS protocols, whereas 3206 are attached to a BIN. We did not analyse 44 samples with sequences shorter than 500 bp, and sequencing failed for 164 specimens.

Details of successfully sequenced voucher specimens, including complete voucher data and images, can be accessed in the Barcode of Life Data Systems (Ratnasingham and Hebert 2007) in the public dataset "DS-LEATNOCT Lepidoptera (Noctuoidea) of Austria" (http://dx.doi.org/10.5883/DS-LEATNOCT). Finally, sequences were submitted to GenBank.

### Data analysis

Identification performance was tested using analytical tools in BOLD systems v. 4.0 (http://www.boldsystems.org). Degrees of intra- and interspecific variation in the DNA barcode fragments were calculated under the Kimura 2 parameter (K2P) model of nucleotide substitution using analytical tools. All species were tested for the presence of a barcode gap, which determines the distribution of distances within one species and to the Nearest Neighbour.

We furthermore tested the congruence of Linnean taxonomy with the recently implemented Barcode Index Number (BIN) (Ratnasingham and Hebert 2013). This system clusters sequences into so-called Operational Taxonomic Units (OTUs), regardless of their previous taxonomic assignment. It is based on a two-stage algorithm that groups the sequences in a cluster and automatically assigns new sequences. All sequences >500 bp and covering some other quality requirements are recorded independently of the project origin and assigned to a BIN (Ratnasingham and Hebert 2013). Ultimately, the BIN system is a tried and tested means of checking the concordance between morpho-taxonomically based species determinations and COI sequence data.

Neighbour-Joining (NJ) and Maximum-Likelihood (ML) trees were constructed using MEGA 7 (Kumar et al. 2016). Node confidences were assessed using 500 bootstrap replicates.

## Results

### Successfully sequenced species inventory

The most updated faunistic Catalogue of Austrian Lepidoptera includes 686 species of Noctuoidea (Huemer 2013), supplemented by 3 recently discovered species (*Eutelia adulatrix*, *Luperina dumerilii*, *Xestia viridescens*) (Huemer 2016, Wieser 2016). We obtained barcode sequences for 671 species of Austrian Noctuoidea, representing around 97.8% of the currently known fauna and belonging to the following families: Noctuidae (478 spp.), Erebidae (139 spp.), Notodontidae (36 spp.), Nolidae (17 spp.), and Euteliidae (1 sp.). The 3223 sequences > 500 bp group as follows: Noctuidae (2341), Erebidae (591), Notodontidae (207), Nolidae (83), and Euteliidae (1).

### Barcode-based species delimitation

A prerequisite for the successful genetic identification of an individual of a species is the presence of a barcoding gap to the genetically most similar species. This means that the maximum genetic distance from an individual within the species A must be smaller than the distance to the closest individual of the nearest species B.

The intraspecific divergence in Austrian Noctuoidea is low, with a mean value of only 0.17% (min. 0% to max. 2.27%) and an average maximum divergence of 0.42% (min. 0% to max. 4.63%), but unknown for 118 species with only singleton sequences.

The interspecific distances to the Nearest Neighbour are significantly higher at an average of 4.95% (min. 0%, max. 14.35%). Out of 671 analysed species, only 13 species (1.9%) could not be reliably identified by their DNA barcode (*Setina aurita*, *Setina irrorella*, *Conisania leineri*, *Conisania poelli*, *Photedes captiuncula*, *Photedes minima*, *Euxoa obelisca*, *Euxoa vitta*, *Euxoa tritici*, *Mesapamaea secalella*, *Mesapamea secalis*, *Amphipoea fucosa*, *Amphipoea lucens*). Only 49 species (7.28%) of the species stock have an interspecific divergence to the Nearest Neighbour of less than 2%. Extensive studies in many groups assume a threshold of approximately 2-3% interspecific divergence as the critical limit to species identification (Foottit et al. 2008, Hausmann et al. 2011a, Zhu et al. 2017). However, whether a barcoding gap exists for individual species pairs has to be examined in each individual case, and for 37 of the 49 species with interspecific divergence <2% we have found a barcoding gap.

In comparison, an analysis of sequences >500 bp of all species and specimens of Austrian Noctuoidea (553 spp.) shows a mean interspecific DNA barcode distance of 10.02% (min. 2.49%, max. 21.95%), whereas in congeneric species the mean distance is 6.06% (min. 0%, max. 16.50%).

### Species delimitation with BINs (Barcode Index Numbers)

671 species of Austrian Noctuoidea representing 3202 records with BINs are assigned to a total of 678 BINs. The vast majority of 649 species is placed into a single BIN (on a national scale), including 13 barcode-sharing species, and there is little evidence of taxonomic mismatches in this group. Twenty-one species are attached to multiple BINs and should be checked for potential cryptic diversity. In this group, only three species of Erebidae (*Coscinia cribraria*, *Setina aurita*, *Setina irrorella*) and one Noctuidae (*Dryobotodes eremita*) have three BINs, whereas 17 species are characterised by two BINs. Taking into account all known BINs at European level, the number of species with multiple BINs increases to 55 species, with the above-mentioned Erebidae showing the highest amount of BINs (ranging from 4 to 9) (Table 3). All these taxa have to be further analysed in an integrative approach and likely at least some of them represent cryptic diversity (see also below).

The proportion of species with multiple BINs shows a considerable variation between different families, ranging from 0% to 19.4% (on a European scale): Notodontidae (36 spp., 19.4%), Erebidae (139 spp., 7.9%), Noctuidae (478 spp., 7.7%), Nolidae (17 spp., 0%) and Euteliidae (1 sp., 0%). In particular, the high number of multiple BIN species on a continental scale in Notodontidae is surprising and clearly indicates a hitherto underestimated diversity. However, on a national Austrian level the genetic diversity in this family is not reflected.

Sixty-three out of 125 singleton BINs in our study were previously unknown in BOLD and are so far only reported from Austria.

### Discordance of morphology and DNA barcodes

Genetic discrimination with DNA barcodes failed for 6 Austrian species pairs/triplets (including 13 species) due to barcode sharing or overlap.

*Setina aurita* – *Setina irrorella*

*S. aurita* and *S. irrorella* are well documented examples of widespread introgressive hybridization (Trawöger 1991) and cannot be separated by DNA barcodes. In contrast, Ortiz et al. (2017) reported barcode divergences for the species *S. cantabrica* and *S. flavicans* based on a limited number of sequences from Spain. On a European scale, however, these results are incomprehensible and the different species cluster without specific grouping, thus indicating a much more widespread hybridization scenario.

*Amphipoea fucosa* – *Amphipoea lucens*

The species pair is easily separated by structures of the male and female genitalia (Zilli et al. 2005). Interestingly, DNA barcode sharing in the genus *Amphipoea* is not an isolated case and, e.g., is also observed in the morphologically well separated species pair *A. crinaensis* and *A. asiatica* (pers. data).

*Conisania leineri* – *Conisania poelli*

The taxonomy of both taxa was disputed for a long time. Depending on the author, *C. poelli* was treated either as a subspecies of *C. leineri* or as a distinct species. Varga and Ronkay (1991) separated both on species level and described subtle differences in the male as well as in the female genitalia. Hacker et al. (2001) also shared thisopinion. The aforementioned authors furthermore distinguished several subspecies in both taxa, which were mainly defined by phenotype combined with allopatric distribution patterns. The small differences in the genitalia and the distinct coloration of the different subspecies could be an indication for a single, widespread species with fragmented distribution and polymorphy in wing coloration, which could depend on different climatic and habitat characteristics. Summarising, the subtle differences in morphology and completely identical barcodes on species level indicate possible taxonomic oversplitting and we suggest further integrative studies to solve this problem.

*Euxoa obelisca* – *Euxoa vitta* – *Euxoa tritici*

These three taxonomically undisputed species of a notoriously difficult genus are well separated by phenotypical appearance. However, interspecific divergence in DNA barcodes is very low with 0.15%, similar to intraspecific variation with at most 0.17%, and identification with DNA barcodes consequently fails.

*Photedes captiuncula* – *Photedes minima*

The species pair is easily recognizable by external morphology and, according to Hausmann et al. (2011b), also shows a minimum pairwise distance of 0.64% in DNA barcodes. However, based on supplementing sequences, both species are not unequivocally separated by their barcodes, which can partially overlap.

*Mesapamea secalis* – *Mesapamea secalella*

This species pair is usually easily separated by genitalia morphology (Zilli et al. 2005). Analysis of 15 *M. secalis* and 5 *M. secalella* resulted in a very low minimum pairwise distance of 0.31% in DNA barcodes between both species. However, diagnostic substitutions in the barcode region, as stated by Hausmann et al. (2011b) for German samples, could not be confirmed. BOLD analytical tools only fixed 3 partial characters for *M. secalis* and 73 for *M. secalella*. Considering the comparatively large intraspecific divergence of maximum 0.81% in *M. secalis* and 0.62% in *M. secalella* in our sample and the DNA barcode overlap in further yet unverified samples from BOLD, we consider supplementing studies necessary. A third taxon, viz. *Mesapamea remmi*, is currently considered as a likely hybrid (Hausmann et al. 2011b).

A similar case is found in the genus *Schrankia*, with *S. intermedialis* considered as a hybrid of *S. costaestrigalis* and *S. taenialis* and barcode sharing with the latter (Fibiger et al. 2010).

On a European scale, several additional taxa cannot be identified from COI sequences (Table 2). However, these genetically cryptic species pairs/triplets are so far only known from a single species in Austria.

### Deep intraspecific splits - potential cryptic diversity

Fourteen species with a maximum intraspecific distance >2% were found, a threshold which is often used for insect species delimitation (Table 1). The cases which could include cryptic diversity are discussed additionally.

*Bryophila ereptricula*

**Figure 1.**
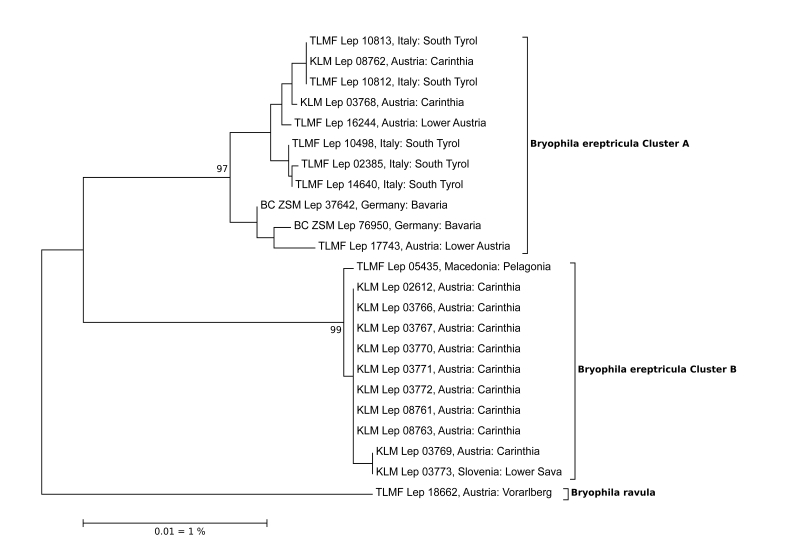
NJ-Tree of *Bryophila ereptricula*. Source: boldsystems.org.

This taxon shows a particularly high maximum intraspecific distance of 4.43%. A NJ analysis results in two well-separated clusters, one with a south-eastern European distribution pattern and the other restricted to the western part of the continent. Despite the deep barcode splits, a first examination of the male genitalia shows no obvious diagnostic features supporting two separate species (Fig. 1). The eastern one (Greek population) was described as *B. ereptricula hellenica* due to differences in the coloration of the wings. This subspecies was synonymised in a revision of this group (Fibiger et al. 2009).

*Parastichtis suspecta*

**Figure 2.**
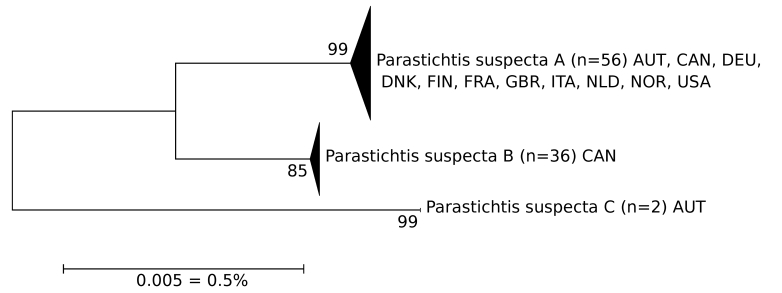
NJ-Tree of *Parastichitis suspecta*. The scale only refers to the branches between the species. The width of the triangles represents the number of samples, the depth the relative genetic variation within the cluster (2× scale). Source: boldsystems.org.

The barcodes of this taxon are divided into three well-separated clusters: one with a large number of samples from most of Europe and Canada, one restricted to Canada and a third consisting of two samples from eastern Austria (Fig. 2). The dissection of the male genitalia of specimens from two clusters occurring in Austria did not give evidence of two different species. However, further integrative research is needed to reach a conclusion.

*Mythimna ferrago*

**Figure 3.**
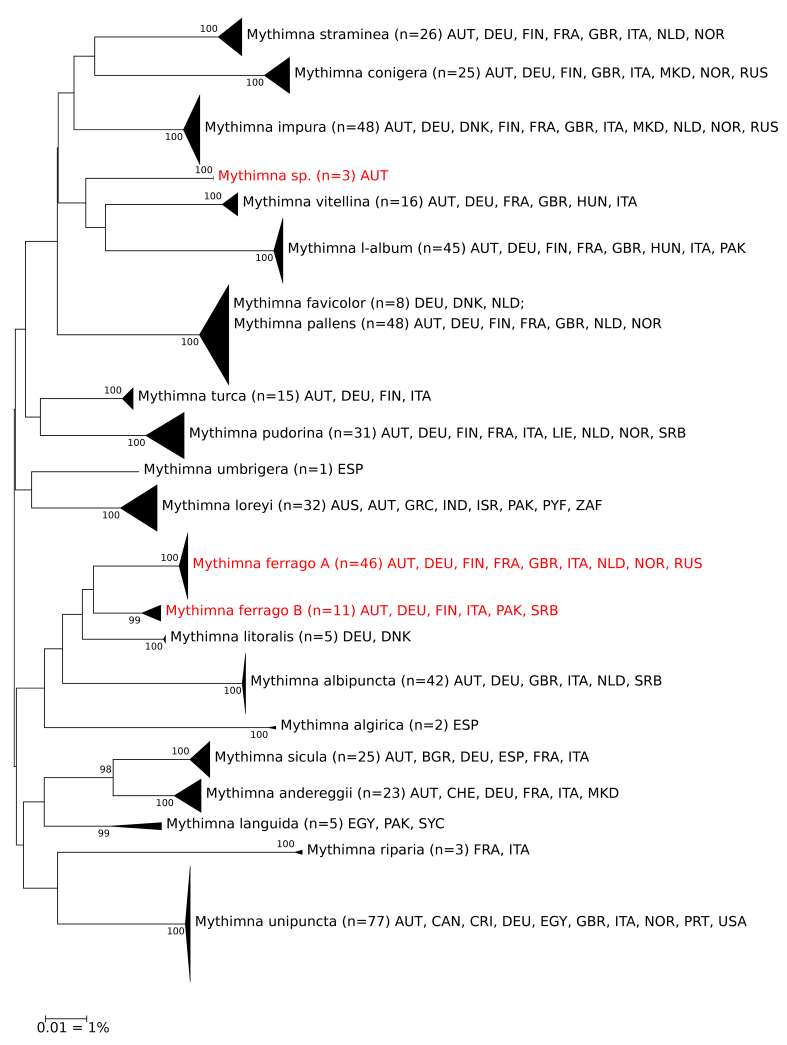
NJ-Tree of European species of the genus *Mythimna*. Unresolved cases highlighted in red; abbreviations of countries follow ISO 3166-1 alpha-3. The scale only refers to the branches between the species. The width of the triangles represents the number of samples, the depth the relative genetic variation within the cluster (2x scale). Source: boldsystems.org.

*Mythimna ferrago* shows two different clusters; both do not reflect a geographical pattern (Fig. 3).

*Enargia paleacea*

**Figure 4.**
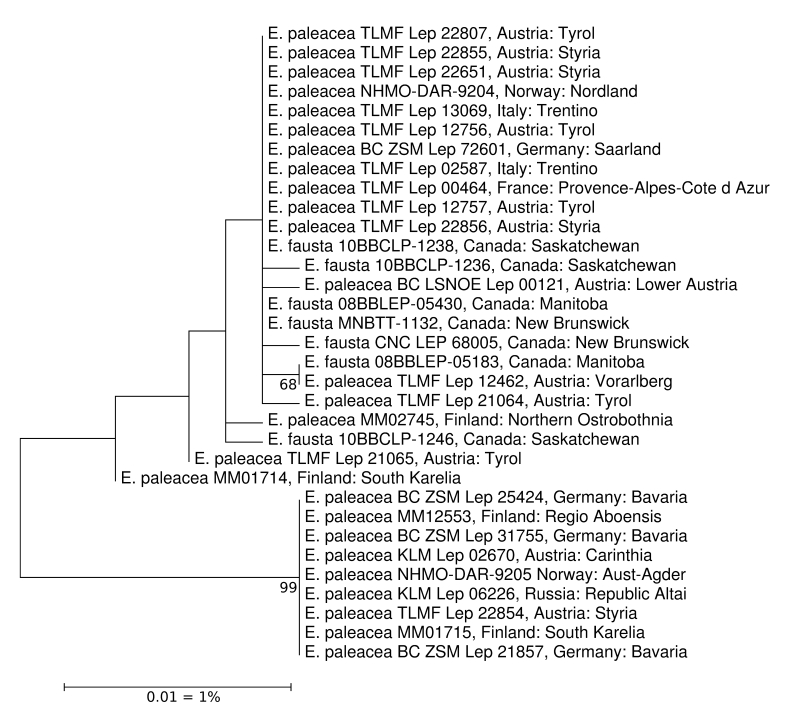
ML-Tree of *Enargia paleace*a and *Enargia fausta*. Source: boldsystems.org.

In his thorough revision of Nearctic *Enargia*, Schmidt (2010) described *E. fausta* as sister taxon of the European *E. paleacea*. Diagnostic characters were identified in subtle morphology, deviating DNA barcodes, and in the distribution area, Nearctic versus Palearctic. It therefore comes as a surprise that we now found two barcode clusters of *E. paleacea* in Europe (and Austria), one of which includes *E. fausta*. We assume from this pattern that *E. fausta* is either a species with Holarctic distribution or a synonym of *E. paleacea*, leaving the identity of specimens in the second cluster unresolved. The problem requires a revision of available names from Europe not yet or insufficiently considered and an in-depth analysis of alleged diagnostic characters of *E. fausta* and *E. paleacea* sensu Schmidt (Fig. 4).

The species *Hypenodes humidalis*, *Rivula sericialis*, and *Coscinia cribaria* show high intraspecific genetic diversity. In all these species, several different clusters, usually without geographic pattern, could be found in DNA barcode sequences. All species remain unexamined and have to be analysed in future in an integrative approach.

### Cases of overlooked species

Three species are currently not identified on species level and belong to additional and hitherto overlooked species for the Austrian fauna, supplemented by a hitherto neglected species pair.

*Hoplodrina* sp.

Already Huemer (2013) found deep splits in DNA barcodes of the widespread *H. octogenaria*, which were interpreted as an indication of possible cryptic diversity. After extensive studies of morphology and applying state-of-the art molecular methods such as ddRadseq, this hypothesis is now well supported and an extensive review including the description of a new species is in preparation (Ronkay et al., in prep.).

*Mythimna* sp.

This remarkable species is phenotypically very similar to the widespread *M. impura*, with which it was initially mixed up. However, DNA barcodes of three simultaneously collected specimens from easternmost Austria (Burgenland, Hackelsberg) are far distant from any of the European *Mythimna* and *Leucania* species. The nearest match to its BIN (BOLD:ADF0473) in BOLD is *Leucania insueta* from North America with a 5.67% distance. Unfortunately, male genitalia morphology often only shows weak or subtle diagnostic characters in the genera *Mythimna* and *Leucania* and an integrative analysis of this problem seems mandatory.

*Aporophyla lutulenta* – *Aporophyla lueneburgensis*

This species pair has subtle differences in the wing pattern as well as in the genitalia features. Therefore, the distribution and the status of these taxa is controversially discussed. Ronkay et al. (2001) separated two species, *A. lutulenta* with an Antlantico-Mediterranean and *A. lueneburgensis* with Ponto-Mediterranean distribution. Orhant (2012) found only one barcode cluster in French specimens and consequently synonymised both taxa. However, in this study, only true *A. luenburgensis* have been sequenced, whereas correctly identified *A. lutulenta*, a species described from Lower Austria, were accidentally not taken into account. It was therefore not a surprise when Haslberger and Segerer (2016) found two separate clusters, indicating species status for *A. lueneburgensis* and *A. lutulenta*. Both these clusters are found in Austria, but further taxonomic work is needed to solve this problem. A taxonomic study focusing on these two taxa is in progress.

*Lygephila* sp.

A first and preliminary analysis of morphological traits in *Lygephila craccae* gives no evidence of cryptic diversity, whereas further genetic analysis of nuclear genes support two different species. Further integrative taxonomic work will be needed to solve this question.

### Cases of likely taxonomic oversplitting

Two widely accepted species pairs/groups with barcode sharing or overlap are considered as likely taxonomic oversplitting since neither DNA barcodes nor morphology convincingly support species status. These cases are counted as a single species in our analyses and are in strong need of thorough, integrative revisionary work (but see also *Conisania* spp. above).

*Euxoa tritici* s.l. – *Euxoa selignis*

This species complex is one of the most controversial taxonomic problems in the European Noctuoidea, including the disputed taxa *Euxoa tritici*, *E. nigrofusca* and *E. eruta*. Following in-depth studies of morphology and DNA barcodes Hausmann et al. (2011b), Mutanen (2005) and Behounek (2011) found no support of a species-complex and the split into three species was consequently not accepted by Huemer (2013). DNA barcoding of Austrian *Euxoa selignis* (nec *segnilis*) leads to strong doubts as to whether (at least) Austrian *Euxoa selignis* are not also *E. tritici*.

*Hadena bicruris* – *Hadena capsincola*

Separation of these two species follows subtle morphological characters and an allopatric distribution pattern (Hacker 1996, Hacker et al. 2001). However, an analysis of 46 barcode sequences in BOLD from distribution areas of both species resulted in the absence of a barcode gap between both alleged taxa, and does not support the two species hypothesis.

## Discussion

Noctuoidea are the most diverse group of Lepidoptera of Austria (Huemer 2013), and with almost 700 species or about 17% of the national fauna, are highly representative for this insect order. The successful sequencing of approximately 98% of the species inventory of Austrian Noctuoidea is therefore *per se* an important contribution to Austria's national DNA barcode library.

98.2% of the successfully sequenced 671 species can be reliably distinguished from other species by their barcode. Similarly, the species differentiation with BINs results in successful delimitation of 96.7% of the species inventory, which are all attributable to a single BIN. Only 12 species cannot be reliably distinguished from the barcode while, conversely, the interspecific divergence to the Nearest Neighbour is >2% in just under 93% of the species. These astonishing similarities with classical, morphology-based Linnean taxonomic concepts are confirmed by other studies, dealing with a larger set of a regional fauna, e.g., Hausmann et al. (2011b) found diagnostic DNA barcodes for 99% of Bavaria's larger butterflies and moths. Huemer and Hebert (2016) recognised a correct identification from DNA barcodes for 97% out of ca. 2500 species from an alpine transect in South Tyrol and Tyrol.

A similar high proportion of diagnostic DNA barcodes can be found in many other animal groups in Europe. Overall, most studies demonstrate a high degree of effectiveness of DNA barcoding for reliable genetic species delineation, assuming high standards of morphological determination of the samples tested. Thus, Schmid-Egger et al. (2019) were able to differentiate 99% of the species via DNA barcode divergences in several Hymenopteran families. 92.5% of more than 3500 beetle species from Germany and neighbouring regions could be unambiguously identified with DNA barcodes (Hendrich et al. 2014). Similar results are reported by Morinière et al. (2017) for aquatic insects (Ephemeroptera, Plecoptera, Trichoptera), with 89.5% of the species assigned to a single BIN, while most of the multiple BIN species were ultimately genetically determinable due to the uniqueness of the BINs. However, in some insect orders, such as the Orthoptera, species delineation by means of DNA barcodes is more challenging due to, e.g., widespread introgression, which significantly reduces the effectiveness of the method (Hawlitschek et al. 2017). It is one of the shortcomings of DNA barcoding that sequences of a single mitochondrial gene fragment are unsuitable to resolve introgression of mitochondrial DNA as a result of hybridizations (Hebert et al. 2003a, Hebert et al. 2003b), incomplete lineage sorting of mitochondrial haplotypes, occurrence of nuclear mitochondrial pseudogenes, or the influence of endosymbiotic bacteria such as *Wolbachia*. However, in Noctuoidea these problematic cases seem to be rare, which ultimately contributes to a very high success rate in genetic species discrimination. Not surprisingly, Ortiz et al. (2017) found diagnostic DNA barcodes for a complete set of 160 Iberian Erebidae (Noctuoidea). A much more comprehensive, continental study of Noctuoidea from North America, covering 1541 species, revealed increasing problems with a higher area covered, but here too 90% of the species could be indisputably distinguished by their DNA barcodes, even 95.6% on a provincial basis (Zahiri et al. 2014). However, this high DNA barcode performance in Noctuoidea cannot be transferred one-to-one to other groups of Lepidoptera, and is, e.g., significantly lower in Austrian butterflies (Huemer and Wiesmair 2017).

The few cases of detected discordance between DNA barcodes and Linnean taxonomy deserve special attention in future studies. Possible cases of introgression are found only as rare exceptions in 6 species pairs/triplets. Similarly, possible cryptic diversity seems to be rare and mainly includes the 15 species with relatively low DNA barcode splits <2% that require further investigations. These species set partially overlaps with the 22 species of Austrian Noctuoidea with 2 or 3 BINs. Remarkable and obviously overlooked, possibly even undescribed species are *Hoplodrina* sp. and *Mythimna* sp. Conversely, two cases of likely taxonomic oversplitting could be identified.

## Conclusions

Insect decline is currently a public issue and indeed many studies indicate that we are in the midst of a dramatic biodiversity crisis that seriously affects insects (Sánchez-Bayo and Wyckhuys 2019). However, long-term data sets on the phenomenon of insect decline are scarce and monitoring programs the exception (Segerer and Rosenkranz 2018). At the same time, a considerable decline in the number of experts in this animal class has been observed (Frobel and Schlumprecht 2016), with all the resulting risks for taxonomy, agriculture and forestry, nature conservation, etc. Genetic species identifications by means of DNA barcoding, in our opinion, can provide assistance in mitigating the feared increasing deficit of taxonomic expertise in the future. A prerequisite, however, is the reliable delineation of species by unique gene sequences and, consequently, the development of DNA barcode reference libraries. The DNA barcode study of Austrian Noctuoidea is at the same time a contribution to a national DNA barcode library and proof of the effectiveness of genetic species delineation.

## Figures and Tables

**Table 1.** Species with a maximum intraspecific distance >2%.

**Species**	**Sample size**	**Max. intraspecific distance**
*Parastichtis suspecta*	8	4.63
*Bryophila ereptricula*	12	4.43
*Coscinia cribraria*	7	4.10
*Mythimna ferrago*	6	4.10
*Dryobotodes eremita*	8	3.64
*Eilema sororcula*	6	3.29
*Hypenodes humidalis*	6	2.33
*Rivula sericealis*	6	2.33
*Setina aurita*	9	2.33
*Setina irrorella*	5	2.33
*Enargia paleacea*	11	2.32
*Hecatera dysodea*	4	2.17
*Sideridis lampra*	4	2.02
*Euxoa tritici*	10	2.01

**Table 2.** BIN-sharing Austrian species of Noctuoidea with multiple BINs on the European level and barcode sharing/overlap (alphabetical arrangement; *species not reported from Austria).

**Species**	**BIN sharing**	**Barcode overlap**
*Agrochola nitida*/ A. pistacinoides*/ A. dujardini*	yes	yes
*Agrotis fatidica*/ A. proverai*	yes	no
*Agrotis vestigialis*/ A. sabulosa*	yes	no
*Amhipoea fucosa*/ *A. lucens*	yes	yes
Apamea maillardi / A. schildei*	yes	yes
*Cerura vinula*/ C. iberica*	yes	yes
*Chersotis margaritacea*/ C. cyrnea*	yes	no
*Conisania leineri*/ *C. poelli*	yes	yes
*Cryphia algae*/ C. ochsi*/ C. pallida*	yes	no
*Diachrysia stenochrysis*/ *D. chrysitis*	yes	no
*Dichagyris forcipula*/ D. celsicola*	yes	yes
*Euxoa oblisca*/ *E. vitta*/ *E. tritici*	yes	yes
*Griposia aprilina*/ G. wegneri*/ G. skyvai*/ G. bouveti*	yes	yes
*Hadena capsincola*/ *H. bicruris*/ H. atlantica*/ H. azorica*	yes	yes
*Lacanobia splendens*/ *L. oleracea*	yes	no
*Mesapama secalis*/ *M. secalella*	yes	yes
*Mniotype adusta*/ M. bathensis*	yes	yes
*Noctua pronuba*/ N. atlantica*	yes	no
*Nola aerugula*/ N. holsatica*	yes	yes
*Photedes captiuncula*/ *P. minima*	yes	yes
*Setina aurita*/ *S. irrorella*	yes	yes
*Shargacucullia thapsiphaga*/ S. caninae*	yes	yes
*Xestia rhaetica*/ X. fennica*	yes	yes

**Table 3.** Austrian species of Noctuoidea with multiple BINs in Austria and on the European level (likely cryptic species not included).

**Taxa**	**BINs Austria**	**BINs Europe**
*Setina irrorella*	3	9
*Coscinia cribaria*	3	8
*Setina aurita*	3	4
*Dryobotodes eremita*	3	3
*Acronicta megacephala*	2	3
*Agrotis vestigialis*	2	3
*Bryophila ereptricula*	2	2
*Conistra erytrocephala*	2	2
*Eilema sororcula*	2	2
*Epatolmis luctifera*	2	2
*Euchalcia modestoides*	2	2
*Euxoa decora*	2	2
*Hadena magnolii*	2	2
*Hecatera dysodea*	2	2
*Mythimna ferrago*	2	2
*Opigena polygona*	2	2
*Pachetra sagittigera*	2	2
*Parstichtis suspecta*	2	2
*Rviula sericealis*	2	2
*Sideridis lampra*	2	2
*Tholera cespitits*	2	2
*Apamea maillardi*	1	2
*Acronicta euphorbiae*	1	3
*Agrotis bigramma*	1	3
*Bryophila raptricula*	1	3
*Euchalcia variabilis*	1	3
*Ochropleura plecta*	1	3
*Orthosia cerasi*	1	3
*Peridea anceps*	1	3
*Amphipyra tetra*	1	2
*Apamea monoglypha*	1	2
*Arctia villica*	1	2
*Caradrina aspersa*	1	2
*Chelis maculosa*	1	2
*Conisania luteago*	1	2
*Diarsia mendica*	1	2
*Dichygyris forcipula*	1	2
*Drymonia dodonaea*	1	2
*Drymonia querna*	1	2
*Drymonia ruficornis*	1	2
*Eublemma parva*	1	2
*Eugnorisma depuncta*	1	2
*Furcula furcula*	1	2
*Griposia aprilina*	1	2
*Lateroligia ophiogramma*	1	2
*Notodonta tritophus*	1	2
*Omphalophana anthirrhinii*	1	2
*Schinia cardui*	1	2
*Schrankia costaestrigalis*	1	2
*Shargacucullia thapsiphaga*	1	2
*Thaeumetopoea processioneae*	1	2
*Watsonarctia deserta*	1	2
*Xestia lorezi*	1	2(3)
*Xestia ohreago*	1	2
*Xestia speciosa*	1	2(4)

## Hosting institution

Tiroler Landesmuseen Betriebgsges.m.b.H., Natural History Collections, Hall, Tyrol, Austria.
